# Standard and feature tracking magnetic resonance evidence of myocardial involvement in Churg–Strauss syndrome and granulomatosis with polyangiitis (Wegener’s) in patients with normal electrocardiograms and transthoracic echocardiography

**DOI:** 10.1007/s10554-012-0158-6

**Published:** 2012-12-05

**Authors:** Tomasz Miszalski-Jamka, Wojciech Szczeklik, Barbara Sokołowska, Krzysztof Karwat, Katarzyna Belzak, Wojciech Mazur, Dean J. Kereiakes, Jacek Musiał

**Affiliations:** 1 Center for Diagnosis, Prevention and Telemedicine, John Paul II Hospital, ul. Prądnicka 80, 31-202 Kraków, Poland; 2 Department of Internal Medicine, Jagiellonian University of Cracow, Kraków, Poland; 3 The Christ Hospital Heart and Vascular Center/The Lindner Center for Research and Education, Cincinnati, OH USA

**Keywords:** Churg–Strauss syndrome, Granulomatosis with polyangiitis (Wegener’s), Cardiac magnetic resonance imaging, Feature tracking strain, Late gadolinium enhancement

## Abstract

The aim of the study was to evaluate the presence and spectrum of cardiac abnormalities identified by cardiac magnetic resonance (CMR) in subjects in clinical remission of Churg–Strauss syndrome (CSS) and granulomatosis with polyangiitis (Wegener’s) (WG) with normal ECG and transthoracic echocardiography (TTE). Eleven (7 females, 4 males, mean age 42.4 ± 9.6 years) CSS and 10 (4 females, 6 males, mean age 45.3 ± 10.9 years) WG patients in clinical remission with normal ECG and TTE underwent CMR. Segmental peak-systolic myocardial strain (ε_ps_) was measured using feature tracking cine-sequence based technique. Left ventricular (LV) ejection fraction, end-diastolic volume and myocardial mass indexes were 66.2 ± 5.8 %, 66.1 ± 6.6 ml/m^2^, and 61.0 ± 8.9 g/m^2^, respectively. No patient showed regional wall motion abnormalities and signs of myocarditis. Nine CSS and 8 WG patients demonstrated decreased segmental longitudinal, circumferential or radial ε_ps_ and myocardial late gadolinium enhancement (LGE) (6 subendocardial, 10 midwall, 8 subepicardial) areas. In CSS and WG subjects with LVLGE lesions the mean LVLGE extent was 2.0 ± 1.6 % and 2.3 ± 1.5 % (*p* = 0.65), respectively. Segmental ε_ps_ was decreased longitudinally (−11.8 ± 5.6 %) for subendocardial LGE, radially (13.7 ± 8.7 %) for subepicardial LGE, and circumferentially (−16.6 ± 4.2 %), longitudinally (−13.2 ± 5.5 %) and radially (18.8 ± 8.1 %) for midwall LGE, if compared to longitudinal (−22.7 ± 5.1 %), circumferential (−23.6 ± 5.6 %) and radial (34.2 ± 15.7 %) ε_ps_ in controls (11 females, 10 males, mean age 43.9 ± 10.5 years) (all *p* < 0.01). Despite clinical remission, normal ECG and TTE, most CSS and WG patients demonstrate decreased segmental ε_ps_ and non-ischemic LGE lesions without signs of myocarditis.

## Introduction

Churg–Strauss syndrome (CSS) and granulomatosis with polyangiitis (Wegener’s) (WG) represent the rare group of antineutrophil cytoplasmic antibodies (ANCA) associated small vessel necrotizing vasculitis, which may affect the heart [1]. Although clinically evident cardiac manifestations in WG and CSS are rare, heart involvement is not uncommon and is associated with poor outcome [1]. A wide spectrum of cardiac abnormalities has been documented in the course of CSS and WG including myocarditis, coronary vasculitis, valvular heart disease, pericarditis and/or rhythm disorders [1]. The frequency of cardiac involvement varies depending on the level of disease activity and the diagnostic test employed. The ECG and transthoracic echocardiography (TTE) are the most often used screening tools for heart evaluation [2]. Recently, cardiac magnetic resonance (CMR) has emerged as a novel non-invasive imaging modality providing comprehensive and accurate evaluation of myocardial function and structure. Moreover, a new feature tracking cine-sequence based CMR technique for strain measurement has been recently developed and validated to provide insight into intrinsic function of myocardial fibers [3, 4]. The prior studies have shown that strain abnormalities are sensitive markers of contractile dysfunction which may precede a decline in ejection fraction (EF) [4]. Moreover, we have reported that global left ventricular strain measured by speckle tracking echocardiography might be used to depict heart involvement in CSS and WG [5, 6]. To date, the use of CMR to detect myocardial abnormalities in small vessel necrotizing vasculitis has been limited to a few studies and several case reports [7–18]. It has been suggested that despite normal ECG and TTE, subclinical myocardial involvement detected by CMR in clinical remission may be common [7, 14]. Therefore, the aim of this study was to evaluate the spectrum of cardiac abnormalities identified by standard CMR in CSS and WG patients with normal ECG and TTE, and to assess the presence of strain abnormalities in this group of patients.

## Methods

### Study population

Twenty-one consecutive patients with CSS and WG in clinical remission were enrolled prospectively. CSS and WG was diagnosed according to the American College of Rheumatology criteria [19, 20]. Clinical remission was defined as Birmingham vasculitis activity scores (version 3) ≤ 1 in subjects with CCS and Birmingham vasculitis activity score for WG ≤ 1 in subjects with WG [21, 22]. Exclusion criteria included: (a) limited WG (b) abnormal ECG (presence of pathologic Q wave, ST segment abnormalities and/or rhythm disorders) (c) abnormal TTE [left ventricular (LV) EF <50 %, segmental wall motion abnormality, moderate/severe valve regurgitation/stenosis and/or pericardial effusion], and (d) contraindication to CMR including MR-incompatible implants or electronic devices, renal insufficiency (creatinine clearance <60 ml/kg/min), inability to perform breath hold and claustrophobia. The cumulative organ involvement was measured using Disease Extent Index (DEI) [23]. ANCA was determined by indirect immunofluorescence technique, and when positive, an enzyme-linked immunosorbent assay (ELISA, Euroimmunn, Germany) was performed to detect proteinase-3 (PR-3) and/or myeloperoxidase (MPO) ANCA. The study was approved by institutional ethics committee. Informed consent was obtained from each patient.

### TTE

TTE was performed using Vivid 7 system (GE Vingmed Ultrasound A/S, Horten, Norway) and analyzed off-line by an independent, experienced viewer, unaware of clinical and CMR data.

### CMR: imaging protocol

Breath-hold, ECG-gated CMR was performed on 1.5 T scanner (Magnetom Sonata Maestro Class, Siemens, Erlangen, Germany) equipped with cardiac phased array coil. Cine and morphologic imaging was performed in LV two-chamber, four-chamber and long-axis apical views as well as in short-axis views encompassing the entire LV and right ventricular (RV) myocardium. Cine imaging was obtained using balanced steady-state free precession gradient echo (slice/gap thickness = 8/0 mm, matrix = 256 × 192, in-plane resolution = 1.4 × 1.4 mm^2^, TR/TE = 39/1.1 ms, flip angle = 59°). Morphologic images were acquired with T2-weighted short tau inversion recovery (STIR) (slice/gap thickness = 8/0 mm, matrix = 256 × 192, in-plane resolution = 1.4 × 1.4 mm^2^, TR depending upon RR interval, TE = 65 ms, flip angle = 180°) and T1-weighted turbo spin echo imaging (slice/gap thickness = 8/0 mm, matrix = 256 × 192, in-plane resolution = 1.4 × 1.4 mm^2^, TR depending upon RR interval, TE = 6.9 ms, flip angle = 180°). T1-weighted imaging was performed before and early after intravenous infusion of 0.1 mmol/kg body weight gadobutrol (Gadovist, Bayer Schering Pharma, Berlin, Germany). Following contrast injection (10 min), late gadolinium-enhancement (LGE) imaging was performed using T1-weighted segmented inversion-recovery pulse sequence (slice/gap thickness = 8/0 mm, matrix = 256 × 192, in-plane resolution = 1.4 × 1.4 mm^2^, TR/TE = 650/4.9 ms, flip angle = 30°, TI to null normal myocardium).

### CMR: image analysis

Cine, T1-weighted pre- and after-contrast, T2-weighted STIR, and LGE images were assessed off-line (MASS Medis, Leiden, the Nedtherlands) by independent observer, blinded to clinical and echocardiographic data using 16 LV and 9 RV segment models [24].

### Cine images

As previously described endocardial and epicardial RV and LV borders were outlined on short-axis images to calculate EF as well as end-diastolic volume (EDV), end-systolic volume (ESV) and myocardial mass, which were indexed to body surface area [24].

### T1-weighted and STIR images

By quantitative analysis mean T2 and T1 signal intensity (SI) (pre- and post-contrast) were measured in (a) individual LV segments (b) entire LV myocardium at mid LV level in short axis view and (c) skeletal muscle (erector spinae/lattisimus dorsi) in the same slice, as previously described [8]. T2 myocardial SI was related to skeletal muscle SI and T2-SI ratio was calculated. Myocardial early gadolinium enhancement (EGE) ratio was calculated as myocardial to skeletal muscle enhancement ratio. Global (i.e. determined in entire LV myocardium) T2-SI ratio ≥2.0 and global EGE ratio ≥4 were considered abnormal and indicative of myocardial edema and myocardial hyperemia/hyperpermeability of capillaries, respectively [25]. By analogy, T2-SI ratio ≥2.0 and EGE ratio ≥4 were considered abnormal for individual LV segments.

### LGE images

LGE images were assessed qualitatively for the presence and location of hyperintense lesions in contrast to hypointense viable myocardium. LGE lesions in LV myocardium were defined as transmural, subendocardial (adjacent to endocardium), midwall and subepicardial (adjacent to epicardium) [25]. Based on visual assessment of distribution they were classified as non-ischemic or ischemic. LGE size was assessed manually with planimetry on short-axis slices, delineating hyper enhanced areas. The volume of LGE lesions was calculated and their extent was expressed as the percentage of myocardial volume.

### Myocardial inflammation

According to Lake Louise Criteria CMR was considered to indicate myocardial inflammation, when ≥2 of following criteria were present: (a) global T2-SI ratio ≥2.0 (b) global EGE ratio ≥4 and (c)≥1 focal non-ischemic LGE lesion [25].

### Myocardial strain analysis

Myocardial strain analysis was performed using Diogenes CMR feature tracking software (TomTec Imaging Systems, Munich, Germany) applied to two-, three- and four-chamber long axis as well as apical, mid and basal short axis cine images. The subendocardial contours were drawn manually at the end-diastolic frame and then automatically propagated throughout the cardiac cycle. The contouring was manually adjusted to delineate endocardium in every segment at each cine frame, if automatic tracking of endocardial border failed. If after contour adjustment the endocardial border was not accurately tracked throughout the entire cardiac cycle, the segment was considered unevaluable. Myocardial strain was measured using feature tracking technique in 4 pixels neighborhood and radial strain was related to automatically detected epicardial border. The segmental longitudinal, circumferential and radial peak systolic strain (ε_ps_) was measured using 16 segment LV model by an independent observer blinded to clinical and other CMR data. If according to consensus of two experienced observers the course of segmental strain curve at systolic phase was considered to be substantially interfered with artifacts the segment was excluded from analysis.

To determine intraobserver variability 10 randomly selected studies were reevaluated 2 month later by the same observer unaware of prior results. To assess interobserver variability the images were evaluated by the second observer blinded to the results obtained by first one. To determine the range of normal segmental ε_ps_ CMR was performed in 10 age- and sex-matched healthy volunteers.

### Statistical analysis

Categorical data are presented as numbers (percentages), while continuous data as mean ± SD or median with interquartile range, where appropriate. The normal distribution was verified using Kolmogorov–Smirnov test. Categorical variables were compared by Fisher’s exact, Chi square or Cochran Q test, while continuous variables by unpaired/paired student *t* test and Wilcoxon rank-sum test, where appropriate. Pearson product-moment correlation was performed to assess relationship between the LVLGE extent/volume and parameters reflecting disease activity. The cut-off value for decreased ε_ps_ was 5th percentile for positive and 95th percentile for negative ε_ps_ values measured in healthy individuals. The subject was classified as having reduced segmental ε_ps_ when ≥2 LV segments demonstrated decreased ε_ps_. Inter- and intraobserver variability of ε_ps_ measurement was assessed using Bland–Altman method. Statistical analyses were performed using SPSS software (version 12.0, SPSS Inc., Chicago, IL, USA) with *p* < 0.05 considered statistically significant.

## Results

### Study group

Eleven CSS patients (7 females, 4 males, mean age 42.4 ± 9.6 years) and 10 WG patients (4 females, 6 males, mean age 45.3 ± 10.9 years) were enrolled (Tables 1, 2). No CSS or WG patient demonstrated heart failure or angina symptoms at enrollment. Three CSS and 2 WG patients had developed clinical cardiac manifestations in course of their disease. Of those, all CSS patients presented with perimyocarditis (one with sudden cardiac arrest), while one WG patient developed myocarditis and one non ST-segment elevation myocardial infarction without obstructive epicardial coronary artery stenoses. Six patients reported cigarette smoking, 9 hypertension, 9 hypercholesterolemia, 1 diabetes mellitus and 1 obesity (body mass index ≥30 kg/m^2^). All patients had normal 12-lead ECG. None of the patients demonstrated LVEF <50 %, segmental wall motion abnormalities, moderate/severe valve regurgitation/stenosis, increased systolic pulmonary artery pressure, or pericardial effusion on TTE. Comparing patients with WG and CSS, the former had higher ANCA titre at the time of enrollment (20 (12.5–70) versus 0 (0–10); *p* = 0.002). Similarly, the maximally measured ANCA titre at prior flares was higher in WG than CSS subjects (200 (80–560) versus 0 (0–10); *p* < 0.001). CRP was similar in both groups at the time of enrollment. Conversely, maximally measured CRP was higher in subjects with WG, than CSS (86.7 ± 55.1 vs 37.0 ± 11.6 mg/l; *p* = 0.009). In CSS the actual and maximally measured blood eosinophilia was 198 ± 128 and 6964 ± 5882 cells/μl (*p* < 0.001), respectively. Comparing WG patients with and without prior clinical cardiac manifestations the differences in actual (20 (20–20) versus 30 (15–100); *p* = 0.59) and maximally measured PR3-ANCA titre at prior flare(s) (180 (40–320) versus 200 (40–800), *p* = 0.68) were insignificant. Similarly, in CSS subjects with and without prior clinical cardiac manifestations the differences in the actual (0 (0–0) versus 0 (0–20), *p* = 0.24) and maximally measured MPO-ANCA titre at prior flare(s) [0 (0–0) vs 0 (0–30); *p* = 0.24] did not reach statistical significance.

**Table 1:** Patient characteristics

	CCS (n = 11) / WG (n = 10)	*p*
Female/male	7/4 / 4/6	0.39
Age [years]	42.4 ± 9.6 / 45.3 ± 10.9	0.30
Time since diagnosis [years]	4.7 ± 2.8 / 4.8 ± 3.9	0.99
*Enrolment*		
Positive cANCA/pANCA	0/3 / 10/2	0.02
ANCA titre^a^	20 (20–30) / 20 (12.5–70)	0.81
PR3-ANCA titre^a^	0 (0–0) / 20 (20–40)	<0.001
White blood cells [10^3^cells/μl]	7.5 ± 3.7 / 7.5 ± 2.9	0.99
Blood eosinophilia [cells/μl]	198 ± 128 / 121 ± 53	0.09
CRP [mg/l]	3.2 ± 2.9 / 5.8 ± 5.3	0.12
Creatinine clearance [ml/min]	92 ± 18 / 101 ± 41	0.52
Positive troponin I	0 / 0	>0.99
Treatment: C/M/Az/Cy	1/0/2/2 / 3/1/0/0	0.20
Treatment: G	11/10	>0.99
*Prior flare(s)*		
Ever positive cANCA/pANCA	0/3 / 10/2	0.02
Max ANCA titre^a^	40 (30–100) / 200 (80–560)	0.16
Max PR3-ANCA titre^a^	0 (0.0–0.0) / 200 (40–320)	<0.001
Max white blood cells [10^3^cells/μl]	15.5 ± 9.7 / 14.6 ± 5.3	0.79
Max blood eosinophilia [cells/μl]	6964 ± 8552 / 227 ± 72	0.02
Max CRP [mg/l]	37.0 ± 11.6 / 86.7 ± 55.1	0.009
Min creatinine clearance [ml/min]	84.7 ± 18.3 / 87.2 ± 40.6	0.86
Ever treated: C	9/10	0.48
Ever treated: G	11/10	>0.99

*Az* azathioprine, *C* cyclophosphamide, *Cy* cyclosporine, *G* glucocorticoids, *M* methotrexate, *Max* maximal, *Min* minimal

^a^median with interquartile range for patients with positive ANCA

**Table 2:** Cumulative organ involvement in subjects with CSS and WG with regard to presence of prior clinical cardiac manifestation

Organ involvement	CSS		WG		All	
	Heart (+) / Heart (−) (n = 3) / (n = 8)	*p*	Heart (+) / Heart (−) (n = 2) / (n = 8)	*p*	CCS / WG (n = 11) / (n = 10)	*p*
Upper respiratory tract	3 / 8	>0.99	2 / 8	>0.99	11 / 10	>0.99
Bronchial asthma	3 / 8	>0.99	0 / 0	>0.99	11 / 0	<0.001
Lung	3 / 7	>0.99	1 / 8	0.20	10 / 9	>0.99
Kidney	0 / 4	0.24	1 / 6	>0.99	4 / 7	0.20
Skin	1 / 6	0.49	0 / 4	0.47	7 / 4	0.39
Heart	3 / 0	0.006	2 / 0	0.02	3 / 2	>0.99
Eye	0 / 0	>0.99	0 / 2	>0.99	0 / 2	0.21
Gastrointestinal tract	0 / 3	0.49	0 / 2	>0.99	3 / 2	>0.99
Peripheral nervous system	1 / 5	0.54	1 / 2	>0.99	6 / 3	0.39
Central nervous system	0 / 1	>0.99	1 / 0	0.20	1 / 1	>0.99
Rheumatic complications	2 / 4	>0.99	0 / 3	>0.99	6 / 3	0.39
Constitutional symptoms	3 / 7	>0.99	2 / 5	>0.99	10 / 7	0.31
Cumulative DEI	9.7 ± 3.1 / 10.4 ± 1.6	0.62	9.0 ± 2.8 / 9.4 ± 2.4	0.89	10.2 ± 1.9 / 9.3 ± 3.2	0.45

### Cardiac magnetic resonance

**Fig. 1:**
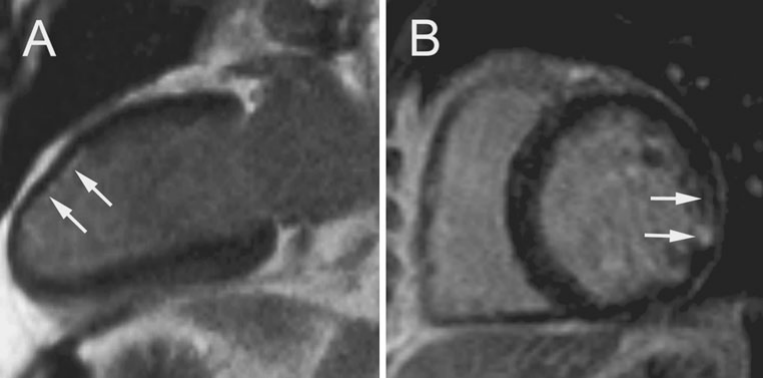
Late gadolinium enhancement (LGE) lesions (arrows) in (**a**) two-chamber apical long-axis view in 59 years old male with granulomatosis with polyangiitis (Wegener’s) (WG) and (**b**) mid ventricular short axis view in 53 years old male with Churg–Straus syndrome (CSS)

LVEF, RVEF, LVEDV index and RVEDV index were 66.2 ± 5.8 %, 61.4 ± 5.7 %, 66.1 ± 6.6 ml/m^2^, and 68.8 ± 7.8 ml/m^2^, respectively. No patient demonstrated abnormal global or regional LV or RV systolic function. Table 3 summarizes CMR findings in CSS and WG subjects. Comparing patients with WG and CSS, the former had higher LV mass index (65.5 ± 8.6 vs 56.9 ± 7.8 g/m^2^; *p* = 0.02). LV and RV LGE lesions were found in 17 and 7 patients, with the mean number of involved segments of 2.3 ± 2.0 and 0.7 ± 1.2, respectively (Fig. 1). In CSS and WG subjects with LVLGE the mean myocardial extent of LVLGE lesions was 2.0 ± 1.6 % and 2.3 ± 1.5 % (*p* = 0.65), respectively. Six LGE lesions were classified as subendocardial, 10 as midwall and 8 as subepicardial. In CSS and WG subjects LGE lesions were found both in the posterior LV portion (inferior and inferolateral wall) and in the interventricular septum (10 vs 8 segments, *p* = 0.77).

**Table 3:** CMR findings in subjects with CSS, WG and controls

CMR parameters	CSS (n = 11) / WG (n = 10)	*p*	All (n = 21) / Controls (n = 21)	*p*
LVEF [%]	68.0 ± 6.1 / 66.0 ± 5.0	0.89	66.2 ± 5.8 / 63.4 ± 3.4	0.13
LVEDV index [ml/m^2^]	66.1 ± 4.0 / 66.1 ± 8.5	0.99	66.1 ± 6.6 / 69.9 ± 8.2	0.15
LV mass index [g/m^2^]	56.9 ± 7.8 / 65.5 ± 8.6	0.02	61.0 ± 8.9 / 49.7 ± 7.8	<0.001
RVEF [%]	63.7 ± 3.7 / 58.9 ± 6.3	0.05	61.4 ± 5.7 / 60.3 ± 7.1	0.60
RVEDV index [ml/m^2^]	68.0 ± 6.9 / 69.6 ± 9.0	0.65	68.8 ± 7.8 / 71.9 ± 8.3	0.28
RV mass index [g/m^2^]	17.8 ± 3.2 / 16.7 ± 1.9	0.36	17.3 ± 2.5 / 17.6 ± 2.2	0.68
Myocardial SI-T2 ratio at mid LV level	1.2 ± 0.2 / 1.3 ± 0.2	0.33	1.3 ± 0.2 / 1.2 ± 0.3	0.37
Myocardial EGE ratio at mid LV level	2.2 ± 0.8 / 2.3 ± 0.3	0.71	2.2 ± 0.6 / 2.2 ± 0.9	0.88
Pericardial effusion	2 / 2	>0.99	4 / 0	0.11
Localized pericardial thickening	2 / 4	0.36	6 / 0	0.02
Decreased ε_ps_ ≥2 LV segments	9 / 8	>0.99	17 / 0	<0.001
Decreased ε_ps_: total (mean) LV segment number	66 (6.0 ± 3.4) / 70 (7.0 ± 3.9)	>0.99 (0.53)	136 (6.5 ± 3.6) / 4 (0.2 ± 0.4)	<0.001 (< 0.001)
*Decreased ε*_*ps*_				
Longitudinally: total (mean) LV segment number	49 (4.5 ± 2.8) / 32 (3.2 ± 2.3)	0.12 (0.28)	81 (3.9 ± 2.6) / 2 (0.1 ± 0.3)	<0.001 (<0.001)
Circumferentially: total (mean) LV segment number	12 (1.6 ± 1.7) / 24 (2.4 ± 1.6)	0.06 (0.32)	36 (2.0 ± 1.7) / 2 (0.1 ± 0.3)	<0.001 (<0.001)
Radially: total (mean) LV segment number	18 (1.1 ± 1.1) / 29 (2.9 ± 1.7)	0.05 (0.01)	47 (2.0 ± 1.7) / 2 (0.1 ± 0.3)	<0.001 (<0.001)
LVLGE: present (segment number)	9 (2.2 ± 1.7) / 8 (2.5 ± 2.3)	0.64 (0.72)	17 (2.3 ± 2.0) / 0 (0.0 ± 0.0)	<0.001 (<0.001)
LVLGE: subendocardial/midwall/subepicardial	3/5/4 / 3/5/4	>0.99	6/10/8 / 0/0/0	<0.001
RVLGE: present (segment number)	4 (0.8 ± 1.0) / 3 (0.6 ± 1.1)	>0.99 (0.80)	7 (0.7 ± 1.2) / 0 (0.0 ± 0.0)	0.009 (0.01)

The extent and volume of LVLGE lesions did not correlate with measured at prior flare(s) maximum level of CRP, ANCA titre, white blood cell count, cumulative DEI and disease duration (all [r] < 0.2; *p* = NS). In CSS patients the extent and volume of LVLGE lesions did not correlate with the maximum level of blood eosinophilia at prior flare(s) (*r* < 0.2, *p* = NS). According to Lake Louise Criteria, no patient demonstrated evidence of myocardial inflammation. Four patients had localized pericardial effusion (>4 mm; all at RV free wall) and six had abnormal localized pericardial thickening.

### CMR feature tracking based strain

**Fig. 2:**
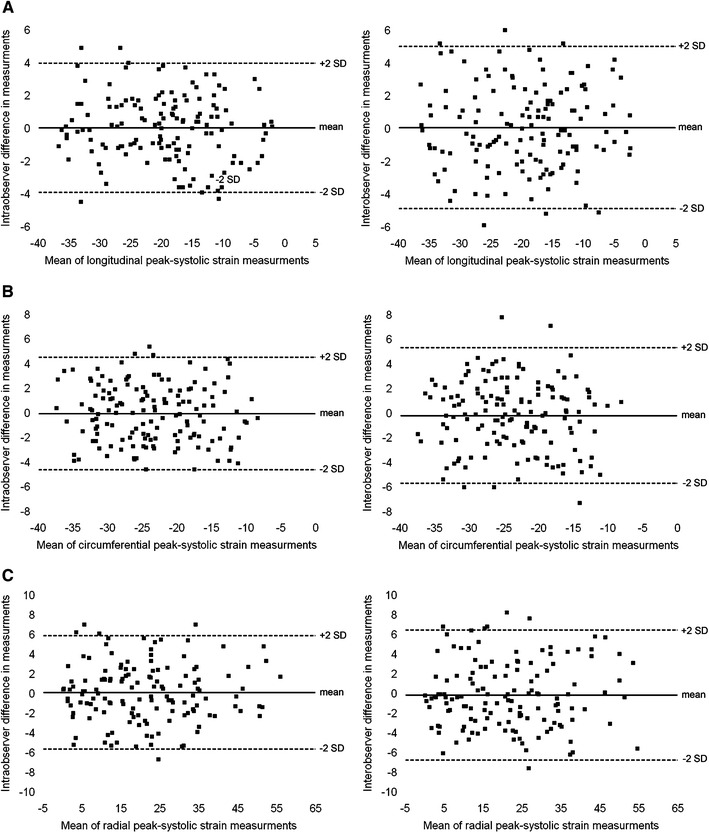
Intra and interobserver reproducibility of segmental longitudinal (**a**), circumferential (**b**) and radial (**c**) peak-systolic strain (ε_ps_) measurements. *SD* standard deviation

**Fig. 3:**
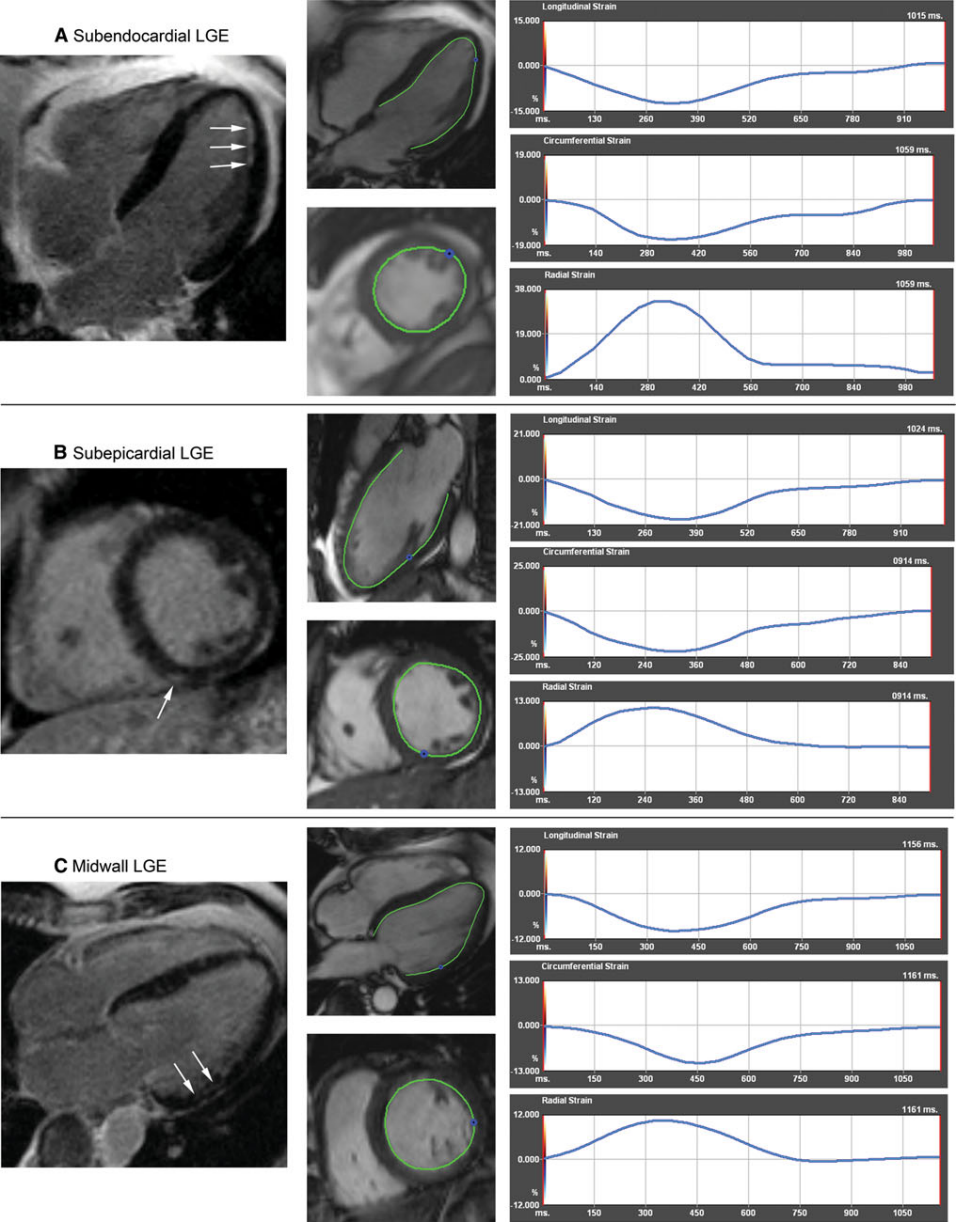
Peak-systolic strain (ε_ps_) with regard to the spatial distribution of late gadolinium enhancement (LGE) lesions (*arrows*) in subjects with Churg–Strauss syndrome (CSS) (**a, c**) and granulomatosis with polyangiitis (Wegener’s) (WG) (**b**). ε_ps_ was decreased longitudinally for subendocardial LGE (**a**), radially for subepicardial LGE (**b**), and circumferentially, longitudinally and radially for midwall LGE (**c**) lesions

Feature tracking based ε_ps_ was measured successfully in all patients. Of 336 LV segments in CSS and WG patients ε_ps_ could not be determined longitudinally in 37(11 %), circumferentially in 31(9 %), and radially in 43(13 %) LV segments. The mean intra- and interobserver bias for segmental longitudinal ε_ps_ was 0.02 ± 2.01 and −0.08 ± 2.27 %, for circumferential −0.06 ± 2.34 and −0.20 ± 2.81 %, and for radial 0.07 ± 2.97 and −0.16 ± 3.38 %, respectively (Fig. 2). In 21 healthy volunteers (11 females, 10 males, mean age 43.9 ± 10.5 years) the mean segmental longitudinal, circumferential and radial ε_ps_ with 5th–95th percentile range was −22.7 ± 5.1 % (−15.9; − 31.3 %), −23.6 ± 5.6 % (−16.1; − 33.0 %), and 34.2 ± 15.7 % (19.9; 64.6 %), respectively. Of those healthy individuals four subjects showed one LV segment with decreased ε_ps_ (two with reduced longitudinal ε_ps_ and two with reduced both circumferential and radial ε_ps_). None of the control group demonstrated decline in ε_ps_ in ≥2 LV segments. Nine CSS and 8 WG subjects (*p* > 0.99) demonstrated decreased ε_ps_ ≥2 LV segments and were considered as having reduced segmental ε_ps_. In patients with CSS and WG decline in at least one of longitudinal, circumferential and radial ε_ps_ was found in 66 and 70 LV segments, respectively (*p* = 0.29). Longitudinal, circumferential and radial ε_ps_ was decreased in 49, 12 and 18 LV segments (*p* < 0.001) in CSS patients, and in 32, 24 and 29 (*p* = 0.48) LV segments in WG patients, respectively. The mean segmental longitudinal ε_ps_ in WG and CSS subjects was lower than in controls (both *p* < 0.01) (Table 4). If compared to controls (a) segments with subendocardial LGE lesions demonstrated decreased longitudinal, but normal circumferential and radial ε_ps_ (b) segments with midwall LGE lesions showed decreased longitudinal, circumferential and radial ε_ps_, while (c) segments with subepicardial LGE areas had decreased radial, but normal longitudinal and circumferential ε_ps_ (Table 4; Fig. 3).

**Table 4:** The mean segmental longitudinal, circumferential and radial peak-systolic strain (ε_ps_) in subjects with Churg–Strauss syndrome (CSS), granulomatosis with polyangiitis (Wegener’s) (WG), controls as well as with regard to the spatial distribution of late gadolinium enhancement (LGE) lesions

Segmental ε_ps_	CSS (n = 11)	WG (n = 10)	Subendocardial LGE (n = 6)	Midwall LGE (n = 10)	Subepicardial LGE (n = 8)	Controls (n = 21)
Longitudinal [%]	−20.2 ± 10.0*	−20.4 ± 9.6*	−11.8 ± 5.6*	−13.2 ± 5.5*	−19.7 ± 2.9	−22.7 ± 5.1
Circumferential [%]	−24.0 ± 7.0	−23.5 ± 7.8	−22.6 ± 3.1	−16.6 ± 4.2*	−21.9 ± 2.8	−23.6 ± 5.6
Radial [%]	30.5 ± 13.3	29.5 ± 17.3	26.8 ± 8.4	18.8 ± 8.1*	13.7 ± 8.7*	34.2 ± 15.7

* *p* < 0.01 when compared to controls

No difference in mean segmental longitudinal ε_ps_ between CSS and WG were found for longitudinal (*p* = 0.91), circumferential (*p* = 0.57) and radial (*p* = 0.65) measurements

## Discussion

This is the first comprehensive CMR evaluation of cardiac involvement in subjects with clinical remission of ANCA-associated small vessel necrotizing vasculitis who have normal ECG and TTE. Despite the lack of clinical, electrocardiological and echocardiographic signs of cardiac disease, heart involvement in CSS and WG is common and the majority of patients demonstrate decreased segmental longitudinal, circumferential and radial ε_ps_ as well as nonischemic LGE lesions, which demonstrate patchy distribution in all myocardial layers and are not accompanied by signs of myocarditis. Interestingly, the decline in segmental longitudinal, circumferential and/or radial ε_ps_ was related to the spatial distribution of LGE lesions, supporting the link between the function of individual myocardial fiber layers and measured systolic deformational parameters.

Although clinical cardiac manifestations in WG and CSS are not common, evidence for subclinical heart involvement during the course of these diseases is growing [7–12]. The wide spectrum and varied prevalence of cardiac abnormalities in small vessel necrotizing vasculitis has been linked to disease activity [1]. Our findings indicate that, despite the lack of cardiac symptoms and clinical remission, the heart is frequently involved in CSS and WG. Importantly, the majority of CSS and WG patients demonstrate subclinical heart abnormalities, which are not detectable by ECG and TTE. Despite normal systolic ventricular function segmental ε_ps_ was frequently decreased and appears to be a sensitive marker of contractile dysfunction. Of note, the decline in ε_ps_ was found for longitudinal, circumferential and radial measurements suggesting the involvement of all myocardial fiber layers. In contrast to WG, CSS patients more frequently showed decreased segmental longitudinal ε_ps_. In addition subjects with CSS, when compared to subjects with WG, tended to have less segments with decreased circumferential and radial ε_ps_. This observation may reflect propensity for subendocardial involvement in CSS, which has been demonstrated in subjects with impaired LVEF by both LGE imaging and endomyocardial biopsy [9, 11, 12]. In general, decreased segmental ε_ps_ might be considered to be an early marker of cardiac involvement in WG and CSS. However due to limited reproducibility more restrictive cut-off values should be proposed and validated using repeated CMR examinations. Interestingly, the reproducibility of segmental ε_ps_ measurements in the present study is better than previously reported by Morton G et al. and Schuster A et al. [26, 27]. This apparent difference may have several explanations. First, unlike these prior reports manual adjustment of endocardial border throughout the entire cardiac cycle was performed in the current study and segments with inaccurate endocardial border tracking or artifacts substantially interfering with the course of strain curve were excluded. Second, if compared to these prior reports, the current study has higher in-plane resolution of SSFP sequence. Finally, the present study utilized 1.5 T scanner, which despite lower signal to noise and contrast to noise ratios produces less field inhomogeneities during cine imaging than 3.0 T scanner employed by Morton G et al. [26, 28]. Importantly, the filed inhomogeneities might affect accurate future-tracking and increase the variability of ε_ps_ measurements, however further studies are needed to assess the issue.

A relatively frequent cardiac abnormality observed in the present study was LGE areas distributed in RV and LV myocardium. As previously reported the lesions were present in all myocardial layers (subendocardial, midwall, subepicardial) both in the posterior LV portion and interventricular septum [8, 10–12, 14, 15]. Interestingly, these LGE areas were not associated with CMR signs of myocardial edema or hyperemia/hyperpermeability of capillaries, which suggests the presence of healed rather than necrotic lesions. The presence of myocardial inflammation has been previously demonstrated during the course of active CSS and WG and the resolution of CMR evidence of myocarditis has been reported in patients treated for disease flare [8, 9, 14]. In this context, the CMR signs of myocarditis in the present study may have resolved as patients reached clinical remission. In the current report the decline in individual segmental ε_ps_ was related to the spatial distribution of LGE lesions. Our data clearly shows that subendocardial LGE lesions correspond with decreased longitudinal ε_ps_, midwall LGE lesions with decreased longitudinal, circumferential and radial ε_ps_, and subepicardial LGE lesions with decreased radial ε_ps_. These associations may reflect cardiac fiber orientation in each myocardial layer and support the mechanistic link between function of individual myocardial fiber layers and strain values [29].

Prior studies have demonstrated variable prevalence and extent of LGE myocardial lesions in WG and CSS. These discrepancies may be attributed to a different clinical profile of studied groups and/or different imaging protocols. Wassmuth et al. [9]. showed that in CSS subjects with clinical suspicion of cardiac involvement, LGE lesions were frequent and had larger extent, than those observed in the present report. Similarly, we have previously found in CSS, that LVEF <50 % is usually associated with a higher number of LV segments with LGE and that patients with severely reduced LVEF often have extensive subendocardial LGE involvement [12–14]. Conversely, a lower prevalence of LGE lesions has been reported in a study of Mavrogeni et al. [10]. who performed LGE imaging, but not cardiac function assessment in WG and CSS subjects with no cardiac symptoms and normal ECG. The apparent discrepancy with the present study may have several explanations. First, Mavrogeni et al. [10]. evaluated CSS and WG patients with both lower disease extent and shorter disease duration. Second, their patients had no history of cardiac disease (versus 5 such patients in the current series). Finally, the signal intensity threshold to delineate LGE areas set by Mavrogeni et al. [10]. was higher than that used in studies of myocarditis, and thus potentially reducing the ability for LGE detection [30].

### Study limitations

First, the study cohort is small and these results should be confirmed in a larger patient cohort. Second, because of the relative contraindication to CMR, patients with low creatinine clearance were excluded and the results may not be applicable to a broader population of CSS and WG patients. Third, we have not assessed ε_ps_ by TTE, what might elucidate its role to depict early cardiac involvement on a segmental basis and determine the potential superiority of CMR technique. Fourth, an assessment of myocardial velocity, displacement and strain rate, which is available using Diogenes CMR feature tracking software, was not performed. Fifth, myocardial perfusion abnormalities were not evaluated using CMR. The prior studies and case reports have shown that first-pass perfusion might detect subendocardial perfusion abnormalities in CSS and WG subjects who present clinical cardiac manifestation and/or active disease. However, the value of first-pass perfusion CMR technique to detect myocardial involvement in subjects with inactive CSS or WG, normal ECG and TTE has not been determined [16–18]. Finally, although endomyocardial biopsy was not performed to exclude myocarditis, this technique has relatively poor diagnostic accuracy due to patchy myocardial involvement in CSS and WG.

## Conclusions

Despite clinical remission, normal ECG and TTE, the heart is frequently involved in WG and CSS. Most patients demonstrate decreased segmental longitudinal, circumferential and radial ε_ps_ as well as nonischemic LGE lesions with patchy distribution in all myocardial layers. These lesions are not accompanied by signs of myocarditis and are not reflected by abnormalities during routine ECG and TTE assessment. In WG and CSS CMR may be the most sensitive, non-invasive diagnostic technique to detect cardiac involvement and feature tracking cine-sequence based CMR strain analysis allows for early detection of myocardial contractile impairments, which may correspond with the spatial distribution of LGE lesions.
